# Using Stochastic Spiking Neural Networks on SpiNNaker to Solve Constraint Satisfaction Problems

**DOI:** 10.3389/fnins.2017.00714

**Published:** 2017-12-19

**Authors:** Gabriel A. Fonseca Guerra, Steve B. Furber

**Affiliations:** Advanced Processor Technologies Group, School of Computer Science, University of Manchester, Manchester, United Kingdom

**Keywords:** SpiNNaker, constraint satisfaction, spiking neural networks, stochastic search, spiking neurons

## Abstract

Constraint satisfaction problems (CSP) are at the core of numerous scientific and technological applications. However, CSPs belong to the NP-complete complexity class, for which the existence (or not) of efficient algorithms remains a major unsolved question in computational complexity theory. In the face of this fundamental difficulty heuristics and approximation methods are used to approach instances of NP (e.g., decision and hard optimization problems). The human brain efficiently handles CSPs both in perception and behavior using spiking neural networks (SNNs), and recent studies have demonstrated that the noise embedded within an SNN can be used as a computational resource to solve CSPs. Here, we provide a software framework for the implementation of such noisy neural solvers on the SpiNNaker massively parallel neuromorphic hardware, further demonstrating their potential to implement a stochastic search that solves instances of P and NP problems expressed as CSPs. This facilitates the exploration of new optimization strategies and the understanding of the computational abilities of SNNs. We demonstrate the basic principles of the framework by solving difficult instances of the Sudoku puzzle and of the map color problem, and explore its application to spin glasses. The solver works as a stochastic dynamical system, which is attracted by the configuration that solves the CSP. The noise allows an optimal exploration of the space of configurations, looking for the satisfiability of all the constraints; if applied discontinuously, it can also force the system to leap to a new random configuration effectively causing a restart.

## 1. Introduction

Most practical problems and natural phenomena can be abstracted as systems composed of smaller elements interacting with each other, an element being able to assume one of many states and the global configuration of states governed by the nature of the interactions. In practice, each interaction imposes a restriction on the behavior of the units (a constraint). Such a description allows the interpretation of the phenomena as a constraint satisfaction problem (CSP), which is defined by the tuple 〈*X, D, C*〉. Here, *X* = {*x*_1_, …, *x*_*N*_} is a set of *N* variables defined over the respective set of non-empty domains *D* = {*D*_1_, …, *D*_*N*_}, each *x*_*i*_ represents an element of the system which can take *D*_*i*_ possible states. The constraints *C* = {*C*_1_, …, *C*_*m*_} are 〈*S*_*i*_, *R*_*i*_〉 tuples defined over *m* subsets *S* = {*S*_1_, …, *S*_*m*_:*S*_*i*_ ⊆ *X*}, and *k* relations *R* = {*R*_1_, …, *R*_*k*_} (Russell and Norvig, 2009). In general, each *R*_*i*_ is a tuple defined over the Cartesian product of the variable domains, if however, all relations *R*_*i*_ are defined as 2-tuples, the CSP is called binary. With this definition, and without taking into account symmetry considerations, one has on the order of ${\bar{D}}^{N}$ possible evaluations for the values of the set *X*. (Here $\bar{D}$ is the average size of the domains). In the case of a Sudoku puzzle, for example, *X* represents the grid cells, the set *D* consists of the nine possible digits for each cell and *C* defines the game rules. In this case one has 9^81^ possible configurations which after puzzle equivalency reduction define ≈6.67 × 10^21^ possible puzzles (Felgenhauer and Jarvis, 2005).

A solution to the CSP (if it exists) is an evaluation of X that is consistent (satisfies all the constraints *c*_*i*_ in *C*) and complete (includes all variables *x*_*i*_ in *X*). To find such a solution one implements a search algorithm that explores the state space of all these configurations. The strategy of searching the whole state space, known as the brute-force algorithm, quickly becomes unfeasible as *N* increases (e.g., requiring more computing time than the age of the universe; Norvig, 2009), demanding the development of cleverer algorithms. The efficiency of such a computing algorithm is conventionally determined with the definition of its asymptotic time complexity *T*(*n*), expressed as a function of the input size of the problem *n*∝*N* for a particular encoding language (Gary and Johnson, 1979). Notice that for a given problem two different instances of the same size *n* could reveal different performance, so *T* refers to the worst-case complexity. According to Cobham's thesis, an algorithm is conventionally considered efficient if it admits worst-case polynomial time solutions on a deterministic Turing machine (DTM). Such algorithms build up the P complexity class, corresponding to $T\left(n\right)\in O\left(n^{\kappa }\right)$, where κ is determined by the nature of the problem (Cobham, 1965). A broader class, the NP complexity, contains all decision problems for which a proposed solution can be verified in polynomial time (Cook, 1971).

The problem of determining the existence of efficient algorithms for solving every NP problem, known as the P versus NP problem, remains unsolved since its establishment by Cook (1971). When a problem does demand algorithms outside P, it is said to be intractable, and it is a widely held view that this is the case for a large subset of NP. Thus, instances of NP are recognized as very hard problems (Fortnow, 2009), the hardest of which are referred to as NP-Complete, which are NP problems to which any other NP problem can be reduced in polynomial time, hence completeness (Karp, 1972)^1^. If *P* ≠ *NP*, NP-complete problems are tractable only by an ideal non-deterministic version of the Turing machine (NDTM) (Cook, 1971; Karp, 1972; Gary and Johnson, 1979). We can think of Turing machines as abstract devices endowed with a set of rules to act on a string of symbols, such actions depending on both, the machine's internal state(s) and the input symbol(s). While at each computation node a DTM has a specific action to perform (thus defining a computation path) an NDTM can follow a whole family of actions (thus defining a computation tree; Hopcroft et al., 2006). At each computation step, either the NDTM takes an action biased toward configurations that lead to accepting states or it branches executing all of the allowed actions (Maruoka, 2011). In any case, an NDTM is guaranteed to find a solution if it exists. Although the biased action description is unrealistic, the replicative interpretation is only limited by the available space and time resources (increasing resources are needed as the NDTM advances through the computation tree). Despite the apparent impracticability of manufacturing an NDTM, very recently, and based on the replicative properties of the deoxyribonucleic acid (DNA) molecule, Currin et al. (2017) reported the first physical design of the embodiment of an NDTM. The practicability of NDTM remains, however, uncertain in the near future. Therefore, with a high possibility of *P*≠*NP* and no NDTMs available, NP problems stay as a hard task to be tackled. Importantly, the determination of the existence (or not) of solutions for a CSP constitutes an NP-complete problem. Therefore, (1) there are no known efficient algorithms that work for general CSPs, despite the fact that there are polynomial time subcases; and (2) any other NP problem can be expressed as a CSP in polynomial time.

NP-Complete problems find applications in a wide range of fields, from spin glass systems, resources allocation, and combinatorial mathematics, to Atari games and public key cryptography (Gary and Johnson, 1979; Barahona, 1982; Fortnow, 2009; Aloupis et al., 2015). Thus, in the absence of known efficient algorithms for solving general NP problems, and the need for at least an approximate solution, the standard strategy is to find either an adequate heuristic or an approximation algorithm for the particular instances of the given problem. The success of such non-neural strategies makes them ideal for some practical applications. Here, our interest is rather in the way in which biological organisms use neuronal networks to efficiently cope with CSPs, in this case even the limitations found are enlightening i.e., it could be more convenient for an animal to prioritize a nearly-optimal but quick solution, especially if the system is unsolvable. Hopfield and Tank (1985) firstly proposed stochastic analog neural networks to solve decision and optimization problems, they had realized the CSP nature of their previously implemented content addressable memory (Hopfield, 1982), and of the optimization of perceptual inference by Hinton and Sejnowski (1983), both of which used networks of binary neurons. More recently, an alternative approach based on deterministic multistable neural oscillators and synaptic plasticity was proposed (Mostafa et al., 2013). All the neural models above are liable to getting stuck in local minima, a cleaver solution was achieved by enhancing the model of Mostafa et al. (2013) with the use of gamma-band rhythmic oscillations of incommensurable frequencies (not rational multiples of each other) (Mostafa et al., 2015b), which further allowed the network dynamics to stabilize when all constraints are satisfied. The latter gave rise to an event-driven, mixed analog/digital prototype chip of incommensurable oscillators which, bespoken to the distributed nature of CSPs, promises to yield state-of-the-art performance (Mostafa et al., 2015a).

In the middle of the 90s, more biologically plausible versions of neural networks, the SNNs, were demonstrated to present equal or superior computational capabilities than those of analog neurons (Maass, 1995, 1996, 1997). Despite promising advantages, their implementation demands a high computational expense in conventional hardware. Regarding CSPs, Malaka and Buck (2000) achieved an SNN solution of an 8 cities traveling salesman problem (TSP). More than a decade later, Habenschuss et al. (2013) demonstrated that the stationary distribution of a stochastic SNN visits the solution of a hard Sudoku puzzle on average 2% of the time once it acquires a performance where 90% of the constraints are satisfied, and finally Jonke et al. (2016), formalized the application of SNNs to general CSPs, postulating a methodology which allows the shaping of the energy landscape, using a modularity principle, controlling the network dynamics and causing it to visit the solution to the problem.

The models above suggest that the noisy, distributed and asynchronous nature of the brain's processes could be behind its computational properties, contrasting with the conventional trends in commercial computer architectures. The brain itself is constantly facing conflicting situations where it should decide actions that best satisfy a number of constraints (Churchland, 2008). Hence, we can take advantage of the brain-inspired computers (neuromorphics) to design new strategies for solving CSPs and gain understanding about which of such strategies are biologically plausible. Given the NP-complete nature of CSPs, it seems natural to consider the research on SNN-solvers to be at an early stage, with the need for an even deeper exploration of their dynamics. It is the aim of this work to provide a tool for the exploration of high-dimensional networks running in biological real time, facilitating the further evolution of SNN-solvers for CSPs, allowing, for example, the study of the non-Boltzmann and non-Markovian dynamics of the network (Crair and Bialek, 1990; Clarke et al., 2015). For this, we use the Spiking Neural Network Architecture (SpiNNaker), a neuromorphic computer which presents a nice balance between the very large number of neurons it is able to simulate, its energy efficiency and the biological real-time feature of the simulations. Neuromorphic computers are electronic devices emulating the working mechanisms of the brain in the search for alternative models of computation. They aim to overcome the limitations offered by conventional computational architectures especially (but not only) with regard to brain simulations (Mead, 1990; Furber S., 2016; Furber S.B., 2016). Similarly to the prototype chip of incommensurable oscillators of Mostafa et al. (2015a), neuromorphics provide a distributed architecture that resembles that of CSPs. They also share the local nature of the constraint graph in which generally a constraint relates only a few variables. SpiNNaker is a real-time asynchronous, multicast, and event-driven machine (Furber et al., 2013, 2014), features that favor the implementation of stochastic computations. Furthermore, it is designed to compute with spiking neurons, overcoming the computational cost that historically limited implementations of SNNs compared to artificial neural networks. Through the following sections, we are going to show how SpiNNaker is able to implement a stochastic search that solves constraint satisfaction problems (CSP). Besides running in biological time our approach improves previous stochastic SNN implementations with the ability to converge into a stable (long-lasting) solution.

## 2. Materials and methods

### 2.1. From constraint satisfaction problems to spiking neural networks

In order to implement the stochastic search we first need to map our CSP into an SNN. Formally, a spiking neural network can be defined as a set of spiking neurons N, each one with a threshold function θ_*i*_, and with connections between two arbitrary neurons $N_{i}$ and $N_{j}$ established by the set of synapses S⊆NXN. For each element $S_{i,j}\in S$ there is a weight parameter *w*_*i,j*_ and a response function $R_{i,j}:{\mathbb{R}}^{+}\to \mathbb{R}$ (Maass, 1997). In our implementation each neuron $N_{i}$ corresponds to a leaky integrate and fire (LIF) neuron (Stein, 1967). In this model the dynamics of the membrane potential *u* are given by:

(1) $$\begin{array}{l} \tau_{m}\frac{du}{dt}=-u\left(t\right)+RI\left(t\right). \end{array}$$

Here, τ_*m*_ is the membrane time constant, *R* is the membrane resistance and *I* an external input current. Each time *u* reaches a threshold value *u*_*th*_ a spike is elicited; such events are fully characterized by the firing times $\left\{t^{f}|u\left(t^{f}\right)=u_{th}and\frac{du}{dt}{|}_{t=t^{f}}>0\right\}$. Immediately after a spike the potential is reset to a value *u*_*r*_, such that $\underset{t\to {t^{f}}^{+}}{\lim}u\left(t\right)=u_{r}$. In our network synapses are uniquely characterized by ω_*ij*_ and the inter-neural separation is introduced by means of a delay Δ_*ij*_. In biological neurons each spike event generates an electrochemical response on the post-synaptic neurons characterized by $R_{i,j}$. We use the same function for every pair (*i, j*), this is defined by the post-synaptic current:

(2) $$\begin{array}{l} j\left(t\right)=\frac{q}{\tau }{\text{e}}^{-\frac{t-t_{0}}{\tau }}\Theta \left(t-t_{0}\right), \end{array}$$

where *q* is the total electric charge transferred through the synapse, τ is the characteristic decaying time of the exponential function, $t_{0}=t^{f}+\Delta_{ij}$ is the arrival time of the spike and Θ represents the Heaviside step function. The choice of $R_{i,j}$ potentially affects the network dynamics, and although there are more biologically realistic functions for the post-synaptic response, the use of the exponential function in Equation (2) constitutes one of our improvements over the previous studies on CSP through SSNs which used a simple square function.

In an SNN each neuron is part of a large population. Thus, besides the background current *I*(*t*), it receives input from the other neurons, as well as a stochastic stimulation from noisy neurons implementing a Poisson process. In this case, the temporal evolution of the membrane potential (Equation 1) generalizes to:

(3) $$\begin{array}{l} \tau_{m}\frac{d}{dt}u=-u\left(t\right)+R\left[I\left(t\right)+\underset{j}{\sum }\omega_{j}\underset{f}{\sum }j\left(t-t_{j}^{f}\right)+\underset{k}{\sum }\Omega_{k}j\left(t-T_{k}\right)\right] \end{array}$$

where the index *f* accounts for the spike times of principal neuron *j* in the SNN, Ω_*k*_ is the strength of the *kth* random spike, which occurs at time *T*_*k*_, and *J*(.) is the response function of Equation (2). An SNN has the advantage that its microstate ψ_*t*_ = {*n*_1_, *n*_2_…, *n*_*N*_} at any time *t* can be defined by the binary firing state *n*_*i*_∈{0, 1} of each neuron $N_{i}$, instead of their continuous membrane potential *u*_*i*_∈ℝ. Then, the set of firing times $\left\{t_{i}^{f}\right\}$ for every neuron $N_{i}$, or equivalently the set of states {ψ_*t*_}, corresponds to the trajectory (dynamics) of the network on the state space. The simulations in this work happen in discrete time (time step = 1ms), so in practice, ψ_*t*_ defines a discrete stochastic process (e.g., a random walk). If the next network state ψ_*t*_*i*+1__ depends on ψ_*t*_*i*__ but is conditionally independent of any ψ_*t*_*j*__ with *j* < *i*, the set {ψ_*t*_} also corresponds to a Markov chain. Habenschuss et al. (2013) demonstrated that this is the case when using rectangular PSPs and a generalized definition of the network state, the validity of the Markov property for general SNNs could still depend on the dynamical regime and be affected by the presence of a non-zero probability current for the stationary distribution (Crair and Bialek, 1990). Each possible configuration of the system, a microstate ψ_*i*_, happens with certain probability *p*_*i*_ and, in general, it is possible to characterize the macroscopic state of the network with the Shannon entropy (in units of *bits*) (Shannon, 1948):

(4) $$\begin{array}{l} S=-\underset{i}{\sum }p_{i}\log_{2}p_{i} \end{array}$$

and the network activity:

(5) $$\begin{array}{l} A\left(t\right)=\frac{1}{N}\overset{N}{\underset{j}{\sum }}\underset{f}{\sum }\delta \left(t-t_{j}^{f}\right) \end{array}$$

To compute *p*_*i*_ and hence Equation (4) we binned the spikes from each simulation with time windows of 200 ms. In this type of high-dimensional dynamical system, sometimes the particular behavior of a single unit is not as relevant as the collective behavior of the network, described for example by Equations (4, 5).

A constraint satisfaction problem 〈*X, D, C*〉 can now be expressed as an SNN as shown in the pseudo-code of algorithm 1. We can do it in three basic steps: (a) create SNNs for each domain *d*_*i*_ of each variable, every neuron is then excited by its associated noise source, providing the necessary energy to begin exploration of the states {ψ}. (b) create lateral-inhibition circuits between all domains that belong to the same variable. (c) create lateral-inhibition circuits between equivalent domains of all variables appearing in a negative constraint and lateral-excitation circuits for domains in a positive constraint. With these steps, the resulting network will be a dynamical system representation of the original CSP. Different strategies can now be implemented to enforce the random process over states ψ_*t*_ to find the configuration ψ_0_ that satisfies all the constraints. The easiest and proposed way of implementing such strategies is through the functional dependence of the noise intensity with time. The size of each domain population should be large enough to average out the stochastic spike activity. Otherwise, the system will not be stable and will not represent quasi-equilibrium states. As will be shown it is the size of the domain populations what allows the system to converge into a stable solution.

The ensemble of populations assigned to every CSP variable *x*_*i*_ works as winner-take-all circuits through inhibitory synapses between domain populations, which tends to allow a single population to be active. However, the last restriction should not be over-imposed, because it could generate saturation and our network will be trapped in local minimum. Instead, the network should constantly explore configurations in an unstable fashion converging to equilibrium only when satisfiability is found. The random connections between populations, together with the noisy excitatory populations and the network topology, provide the necessary stochasticity that allows the system to search for satisfiable states. However, this same behavior traps some of the energy inside the network. For some problems, a dissipation population could be created to balance the input and output of energy or to control the entropy level during the stochastic search. In general, there may be situations in which the input noise acquired through stimulation can stay permanently in the SNN. Thus, the inclusion of more excitatory stimuli will saturate the dynamics in very high firing rates, which potentially reaches the limit of the SpiNNaker communication fabric. In these cases, inhibitory noise is essential too and allows us to include arbitrarily many stimulation pulses.

We demonstrate in the next section that the simple approach of controlling the dynamics with the stimulation intensities and times of the Poisson sources provides an efficient strategy for a stochastic search for solutions to the studied CSPs.

### 2.2. The spiking neural network architecture (SpiNNaker)

With large CSPs the equivalent SNN becomes computationally too expensive for conventional computers, so one of the important contributions of our work is the implementation of the SNN-solver on a computer architecture especially designed for computations with spiking neurons. Conventional supercomputers physically embody a deterministic universal Turing machine and are designed to do computations transferring a high quantity of data in deterministic, synchronous, repeatable and reliable ways. Although under specif circumstances neuromorphic computers can be described by a DTM, they are devices inspired by the working principles of the brain, which is rather asynchronous and unreliable and thus has additional features. Although conventional machines have achieved impressive performance in automatic computing tasks—in part due to the great progress in miniaturization—when facing the complex inference and cognitive tasks solved naturally by living organisms, biology outperforms them by several orders of magnitude, especially with regard to energy efficiency. We believe that such features can provide advantages in the solution of unsolved problems such as the ones in NP.

Neuromorphic computing was first introduced by Carver Mead in the 1980s, originally intended for analogue very-large-scale integration systems. Almost 30 years after Mead's work and after a decade of parallel efforts, there are but a few very powerful, massively parallel neuromorphic computers: TrueNorth (Merolla et al., 2014), Neurogrid (Benjamin et al., 2014), BrainScaleS (Schemmel et al., 2010), and SpiNNaker (Painkras et al., 2013). The latter is endowed with the ability to model high-dimensional spiking neural networks, low energy requirements, and a multicast communication protocol. It is based on a globally asynchronous and locally synchronous (GALS) multi-core System-on-Chip, being event-driven and able to run in biological time. SpiNNaker is built using a million ARM 968 processor cores (of which 60% are currently available). Each chip on the machine includes 18 processor cores connected by a network on chip (NoC) communication system (Grymel and Furber, 2011; Furber, 2012; Furber et al., 2013, 2014; Goodman et al., 2013; Painkras et al., 2013). This fundamentally different architecture paradigm, besides bespoke design for neurobiology simulations, makes the SpiNNaker system interesting for exploring new implementations of stochastic searches. Here we explore the computing power of the machine for these more general computing problems, exploiting the neuromorphic's ability to overcome the conventional difficulties of dealing with computationally expensive spiking neurons when implemented on conventional clusters and GPUs. In summary: (i) for SpiNNaker spiking neurons are the fundamental modeling units and (ii) it is a machine intrinsically able to implement stochastic computations on hardware. We will show in the next section how these two features bring new opportunities to solve hard CSPs.

**Algorithm 1:** Translation of a CSP into an SNN

*# define the CSP = <X, D, C> through a set of lists*.
X=**list**(variables)
D=**list**(domains)
S=**list**(subsets_of(X))
R=**list**(relations_over(s_i **in** S))
C=**list**(constraints = **tuple** (s_i, r_i))
*#a) create an SNN for each variable with sub-populations for each*
domain.
n = size_of_ensemble
**for** variable x_i **in** X:
**for** domain d_i **in** D:
population[x_i][d_i] = create an SNN with n neurons
noise_exc[x_i][d_i] = create a **set** of noise
stimulation populations.
apply_stimuli(noise[x_i][d_i], population[x_i][d_i])
noise_inh[x_i][d_i] = create a **set** of noise
dissipation populations.
apply_dissipation(noise_inh[x_i][d_i], population[x_i][d_i])
*#b) use inhibitory synapses to activate, on average, a single domain per*
variable
**for** domain d_i **in** D:
**for** domain d_j **in** D
inhibitory(population[x_i][d_i], population[x_i][d_j])
*#c) map each constraint to an inhibitory or excitatory synapse*.
**for** constraint c_i **in** C:
read subset s_i **and** relation r_i **from** c_i
**for** variables x_i **and** x_j **in** s_i:
**for** domain d_i **in** D:
**if** constraint relation r_i <0:
inhibition(population[x_i][d_i], population[x_j][d_i])
**elif** constraint relation r_i >0:
excitation(population[x_i][d_i], population[x_j][d_i])

## 3. Results

In order to demonstrate the implementation of the SNN solver, we present solutions to some instances of NP problems. Among the NP-complete problems, we have chosen to showcase instances of graph coloring, Latin squares and Ising spin glasses. Our aim is to offer a tool for the development of stochastic search algorithms in large SNNs. We are interested in CSPs to gain understanding of the dynamics of SNNs under constraints, how they choose a particular state and their computational abilities. Ultimately, SNNs embedded in neuromorphic hardware are intended for the development of new technologies such as robotics and neuroprosthetics, constantly interacting with both the external devices and the environment. In such applications the network needs to adapt itself to time-varying constraints taking one or multiple decisions accordingly, making the advancement in stochastic searches with SNNs a fundamental requirement for neuromorphics.

### 3.1. Graph coloring

Considering a graph *G* defined by the ordered pair {*V, E*}, with *V* a set of vertices and *E* the set of edges connecting them, the graph coloring problem consists of finding an assignments of k colors to the elements of the graph (either *V*, *E* or both) such that certain conditions are satisfied (Dailey, 1980). In vertex coloring, for example, the colors are assigned to the elements of *V* in such a way that no adjacent nodes (those connected by an edge) have the same color. A particularly useful applications of this problem is the process of register allocation in compiler optimization which is isomorphic to graph coloring (Chaitin, 1982). Regarding time complexity, general graph coloring is NP-complete for *k* > 2. In the case of planar graphs, three-coloring is NP-complete and, thanks to the four color theorem proved by Kenneth Appel and Wolfgang Haken, four-coloring is in P (Appel and Haken, 1989).

**Figure 1:**
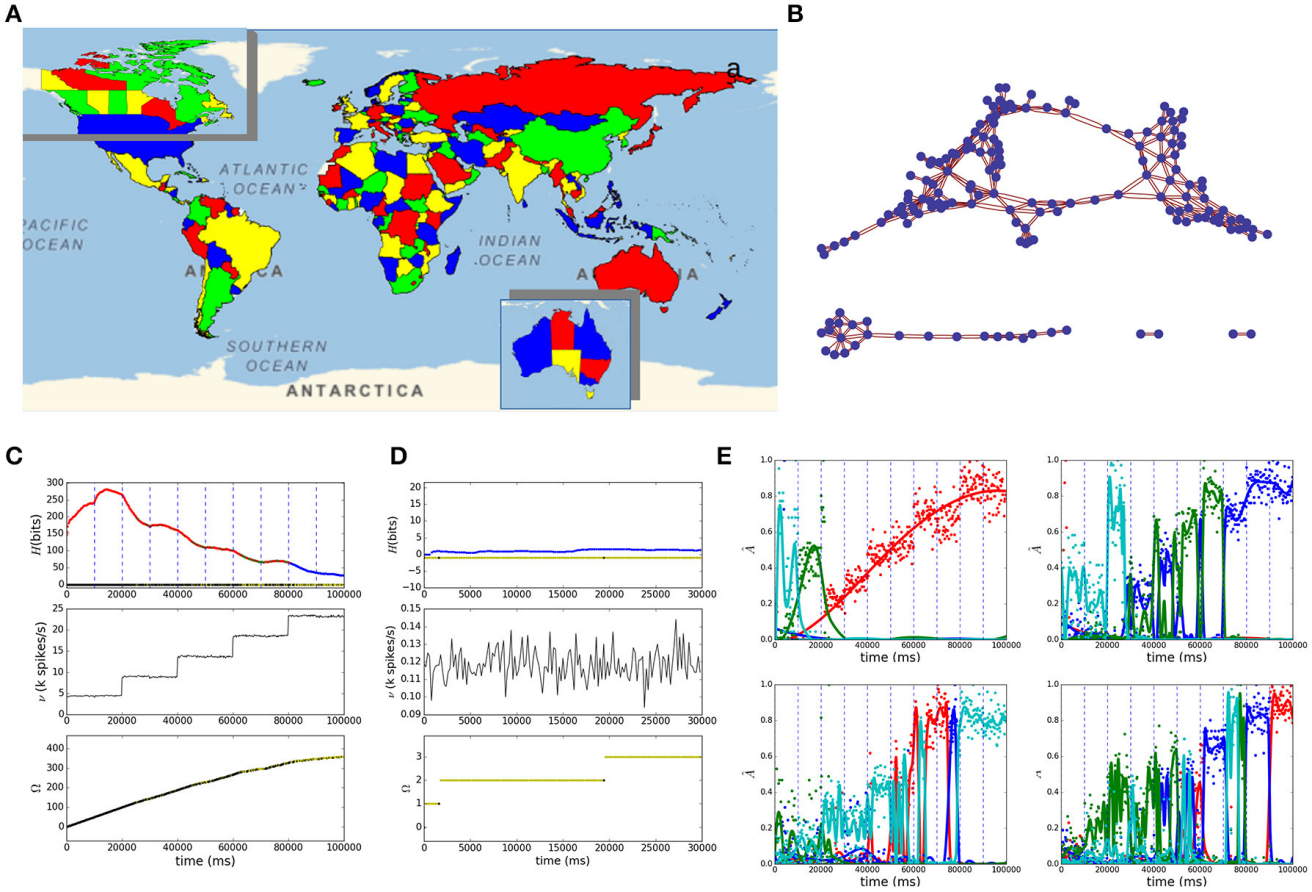
**(A)** Solution to the map coloring problem of the world with four colors and of Australia and Canada with three colors (insets). **(B)** shows the graph of bordering countries from **(A)**. The plots of the entropy *H* (top), mean firing spike rate ν (middle), and states count Ω (bottom) v.s. simulation time are shown in **(C,D)** for the world and Australia maps, evidencing the convergence of the network to satisfying stationary distributions. In the entropy curve red codes for changes of state between successive time bins, green for no change and blue for the network satisfying the CSP. In the states count line, black dots mean exploration of new states; the dots are yellow if the network returns to states visited before. In **(E)** we have plotted the population activity for four randomly chosen CSP variables from **(A)**, each line represents a color domain.

A division of a plane into several regions can be represented by a planar graph, familiar versions of which are the geographic maps. In Figure 1A we show the SNN-solver result of a satisfying four-coloring of the map of the world where colors are assigned to countries such that no bordering countries have the same color. We have followed the list of countries and borders from the United Nations available in Mathematica Wolfram (Wolfram Research, 2017). The corresponding connectivity graph of the world map in Figure 1A is shown in Figure 1B. The insets in Figure 1A, show the results of our solver for three-coloring of the maps of the territories of Australia (bottom-right) and of Canada (top-left). Figures 1C,D show the time dependence of the entropy (top), firing rate (middle), and number of visited states (bottom) for the map of the world and of Australia, respectively. The color code we use in these and the following figures is as follows: red means that the state in the current time bin is different from the one just visited, green represents the network staying in the same state, and blue means that all constraints are satisfied. The dashed vertical lines mark the times at which noise stimulating (blue) or depressing (red) populations began to be active. The normalized spiking activity of the four color populations for four randomly selected countries of the world map is shown in Figure 1E evidencing the competing behavior along the stochastic search. Interestingly, although the network has converged to satisfaction during the last 20 s (blue region in Figure 1C), the bottom right plot in Figure 1E reveals that due to the last stimulation the network has swapped states preserving satisfaction, evidencing the stability of the convergence. Furthermore, it is noticeable in Figure 1D that new states are visited after convergence to satisfiability, this is due to the fact that, when multiple solutions exist, all satisfying configurations have the same probability of happening. Although we choose planar graphs here, the SNN can implement any general graph, hence more complicated P and NP examples could be explored.

#### 3.1.1. Latin squares

**Figure 2:**
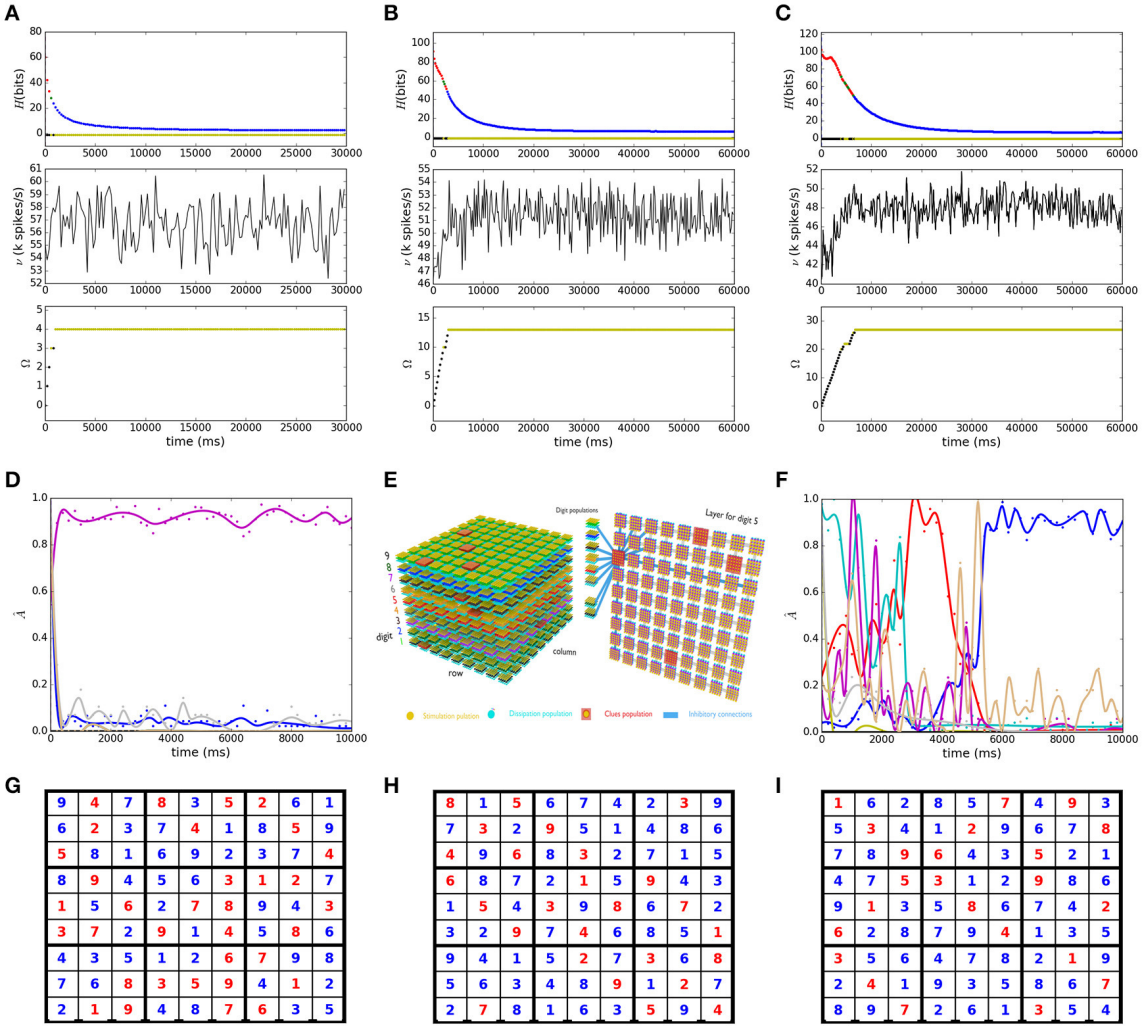
Spiking neural network solution to Sudoku puzzles. **(A–C)** Show the temporal dependence of the network entropy *H*, firing rate ν and states count Ω for the easy **(G)**, hard **(H)**, and AI escargot **(I)** puzzles. The color code is the same as that of Figure 1. In **(G–I)** red is used for clues and blue for digits found by the solver. **(D,F)** Illustrate the activity for a random selected cell from **(A,C)**, respectively, evidencing competition between the digits, the lines correspond to a smoothing spline fit. **(E)** Schematic representation of the network architecture for the puzzle in **(A)**.

A Latin square is defined as an array of *n*×*n* cells in which *n* groups of *n* different symbols are distributed in such a way that each digit appears only once in each row or column. The NP-completeness of completing a partially filled Latin square was demonstrated by Colbourn (1984), and among the useful applications of such a problem, one can list authentication, error-detection and error-correction in coding theory. Here we choose the Sudoku puzzle as an instance of a Latin square, in this case, *n* = 9 and in addition to the column and row constraints of Latin squares, Sudoku requires the uniqueness of the digits in each 3 × 3 sub-grid. We show in Figure 2 the solution to an easy puzzle (Ercsey-Ravasz and Toroczkai, 2012), to a hard Sudoku (Habenschuss et al., 2013) and to the AI Escargot puzzle which has been claimed to be the world hardest Sudoku. The temporal dependence of the network entropy *H*, firing rate ν, and states count Ω is shown in Figures 2A–C, respectively for the easy (Figure 2G), hard (Figure 2H) and AI escargot (Figure 2I) puzzles. In Figure 2E we show a schematic representation of the dimensionality of the network for the easy puzzle (Figure 2G), each sphere represents a single neuron and synaptic connections have been omitted for clarity, the layer for digit 5 is represented also showing the inhibitory effect of a single cell in position (1,3) over its row, column, subgrid and other digits in the cell. In this case, the total number of neurons is ≈37 k and they form ≈86 M synapses.

One major improvement of our implementation with respect to the work of Habenschuss et al. (2013) is the convergence to a stable solution, it is arguably due to the use of subpopulations instead of single neurons to represent the domains of the CSP variables as these populations were required to provide stability to the network. The use of the more realistic exponential post-synaptic potentials instead of the rectangular ones used in Habenschuss et al. (2013) is also reflecting a good performance of the search as shown in the bottom plots in Figures 2A–C, where the solution is found after visiting only 3, 12 and 26 different states and requiring 0.8, 2.8, and 6.6 s, respectively, relating well also with the puzzle hardness. It is important to highlight that the measurement of the difficulty level of a Sudoku puzzle is still ambiguous and our solver could need more complex strategies for different puzzles, for example in the transient chaos based rating of Ercsey-Ravasz and Toroczkai (2012) the “platinum blonde” Sudoku is rated as one of the hardest to solve, and although we have been able to find a solution for it, it is not stable, which means one should control the noisy network dynamics in order to survive the long escape rate of the model presented by Ercsey-Ravasz and Toroczkai (2012). We show in Figures 2D,F the competing activity of individual digit populations of some randomly chosen cell in both the easy and the AI escargot puzzles, the dynamic behavior resembles that of Figure 2 in Ercsey-Ravasz and Toroczkai (2012) when comparing their dynamic solver for this same easy puzzle and the platinum blonde. Further analysis would bring insights into the chaotic dynamics of SNNs when facing constraints.

#### 3.1.2. Ising spin systems

**Figure 3:**
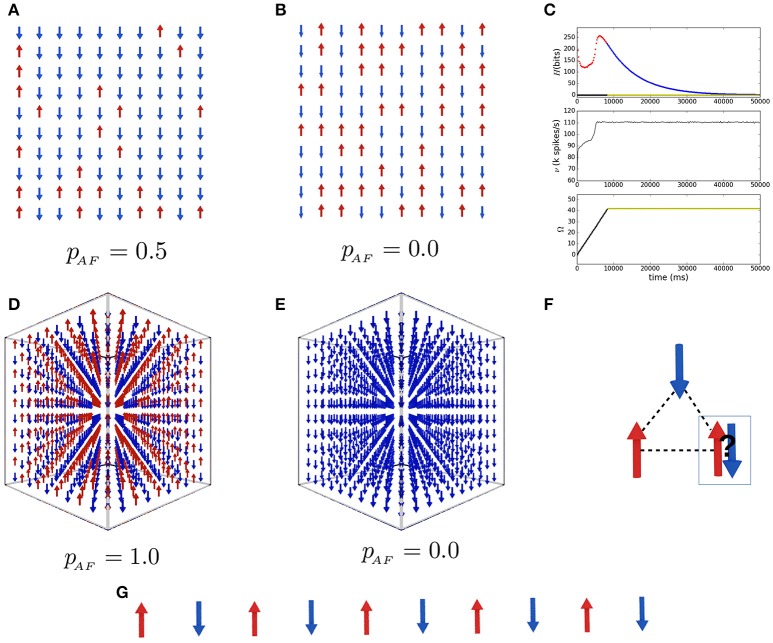
Spiking neural network simulation of Ising spin systems. **(A,B)** Show two 2-dimensional spin glass quenched states obtained with interaction probabilities *p*_*AF*_ = 0.5 and *p*_*AF*_ = 0.1. The results for the 3-dimensional lattices for CSPs of 1,000 spins with ferromagnetic and antiferromagnetic coupling constant are shown in **(D,E)**, respectively. In **(C)** are plotted the temporal dependence of the network entropy, firing rate ν and states count Ω during the stochastic search for the system in **(D)**. **(F)** Illustrates the origin of frustrated interactions in spin glasses. **(G)** Depicts the result for the 1-dimensional chain. The parameters for the SNNs used are shown in Table 1.

For each atom that constitutes a solid, it is possible to define a net spin magnetic moment $\vec{\mu }$ which results from the intrinsic spin of the subatomic particles and the orbital motion of electrons around their atomic nucleus. Such magnetic moments interact in complex ways giving rise to a range of microscopic and macroscopic phenomena. A simple description of such interactions is given by the Ising model, where each $\vec{\mu }$ in a crystal is represented by a spin $\vec{S}$ taking values from {+1, −1} on a regular discrete grid of points {*i, j, k*}. Furthermore, the interaction of the spins $\left\{{\vec{S}}_{i}\right\}$ is considered only between nearest neighbors and represented by a constant *J*_*i,j*_ which determines if the two neighboring spins will tend to align parallel *J*_*i,j*_ > 0 or anti-parallel *J*_*i,j*_ < 0 with each other. Given a particular configuration of spin orientations Ψ, the energy of the system is then given by the Hamiltonian operator:

(6) $$\begin{array}{l} \hat{H}=-\underset{i,j}{\sum }J_{i,j}{\vec{S}}_{i}{\vec{S}}_{j}-\vec{h}\underset{i}{\sum }{\vec{S}}_{i} \end{array}$$

where $\vec{h}$ is an external magnetic field which tends to align the spins in a preferential orientation (Barahona, 1982). In this form each *J*_*i,j*_ defines a constraint *C*_*i,j*_ between the values *D* = {+1, −1} taken by the variables ${\vec{S}}_{i}$ and ${\vec{S}}_{j}$. It is easy to see that the more constraints are satisfied the lower becomes the value of $\hat{H}$ in Equation (6). This simple model allows the study of phase transitions between disordered configurations at high temperature and ordered ones at low temperature. For ferromagnetic *J*_*i,j*_ > 0 and antiferromagnetic *J*_*i,j*_ < 0 interactions the configurations are similar to those in Figures 3D,E for 3D lattices, which correspond to the stable states of our SNN solver when the Ising models for *J*_*i,j*_ > 0 and *J*_*i,j*_ < 0 are mapped to an SNN using algorithm 1 and a 3D grid of 1,000 spins. Figure 3G shows the result for a 1D antiferromagnetic spin chain. It is interesting to note that the statistical mechanics of spin systems has been extensively used to understand the firing dynamics of SNNs, presenting a striking correspondence between their behavior even in complex regimes. Our framework allows the inverse problem of mapping the SNN dynamics to spin interactions. This equivalence between dynamical systems and algorithms has largely been accepted and we see an advantage in computing directly between equivalent dynamical systems. However, it is clear that the network parameters should be adequately chosen in order to keep the computation valid.

If instead of fixing *J*_*i,j*_ to some value *U* for all spin pairs {(*i, j*)} one allows it to take random values from {*U*, −*U*} with probabilities *p*_*AF*_ and *p*_*FM*_, it will be found that certain interactions would be frustrated (unsatisfiable constraints). Figure 3F illustrates the frustration with three antiferromagnetic interacting spins in a way that any choice of orientation for the third spin will conflict with one or the other. This extension of the Ising model when the grid of interactions is a random mixture of AF and FM interactions was described by Edwards and Anderson (1975). The model is the representation of the spin glass systems found in nature, these are crystals with low concentrations of magnetic impurities which, due to the frustrated interactions, are quenched into a frozen random configuration when the temperature is lowered (at room or high temperatures the magnetic moments of a material are constantly and randomly precessing around their average orientation). The statistical analysis of those systems was fundamental for the evolution of artificial neural networks and machine learning. Furthermore, the optimization problem of finding the minimum energy configuration of a spin glass has been shown to be NP-complete by Barahona (1982). The quenching of the grid happens when it gets trapped in a local minimum of the state space of all possible configurations. In Figures 3A,B we show a quenched state found by our SNN with *p*_*AF*_ = 0.5 and *p*_*AF*_ = 0.1, respectively. A spin glass in nature will often be trapped in local minima and will need specific temperature variations to approach a lower energy state; our SNNs replicate this behavior and allow for the study of thermal processes, controlling the time variation and intensity of the excitatory and inhibitory stimulations. If the underlying stochastic process of such stimulations is a good representative of heat in solids, they will correspond to increase and decrease of temperature, respectively, allowing, for example, the implementation of simulated annealing optimization. Figure 3C shows the time evolution of the entropy, firing rate and states count for the antiferromagnetic 3D lattice of Figure 3D, similar plots but converging to unsatisfying states are found for the spin glasses in Figures 3A,B. In the case of the ferromagnetic lattice in Figure 3E with a very low noise, the network immediately converges to a solution, if the noise is high, however, it is necessary to stimulate the network several times to have a perfect ordering. This is because more noise implies more energy to violate constraints, even in nature magnetic ordering is lost at high temperatures.

## 4. Discussion

**Figure 4:**
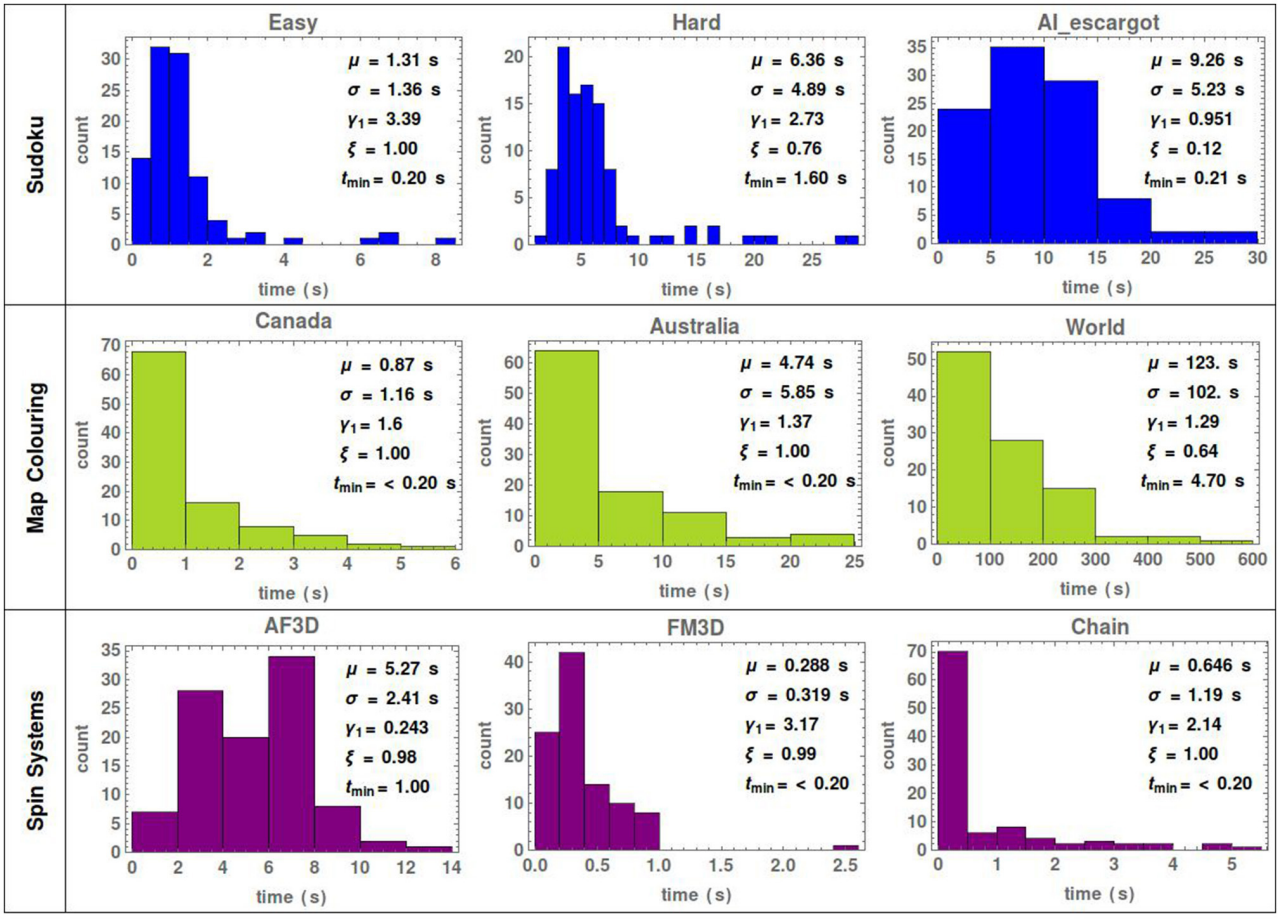
Histograms of the convergence time to a solution for the Sudoku, map coloring and spin system problems of Figures 1–3. For each histogram data from 100 simulations were used. The mean μ, standard deviation σ, skewness γ_1_, success ratio ξ and the best convergence time *t*_*min*_ are indicated for each problem. The success ratio is defined as the number of times the simulation converged to satisfaction over the total number of simulations.

The examples of the last section show the basic features of the stochastic search and the use of the entropy, firing rate and the number of states to track the behavior of the network. In order to evaluate the performance of the search, we have performed a series of runs for each simulation until the network has been successful 100 times. The histograms of the corresponding convergence times for each example are shown in Figure 4, displaying also the mean μ, standard deviation σ, skewness γ_1_, success ratio ξ (defined as the number of times the simulation converged to satisfaction over the total number of runs) and the best convergence time *t*_*min*_ of each underlying distribution. The dimensions of the SNNs and simulation parameters for the three CSPs shown here are summarized, respectively in Tables 1, 2.

**Table 1:** Network sizes of the SNN solvers of the CMP, Sudoku, and Spin Systems.

**Network parameters**				
**CSP**	**Number of neurons**	**Number of synapses**	**Populations (number of variables)**	**Sub-populations (domain size)**
World CMP	212,400	14,422,300	193	4
Australia CMP	450	22,920	7	3
Canada CMP	810	39,480	13	3
Sudoku easy	36,675	86,154,125	81	9
Sudoku hard	36,675	86,154,125	81	9
AI escargot	36,675	86,153,250	81	9
AF ring	1,050	975,500	10	2
Spin 2D lattices	10,050	2,160,000	100	2
Spin AF 3D lattices	100,050	31,050,000	1,000	2
Spin FM 3D lattices	100,050	31,050,000	1,000	2

**Table 2:** Simulation parameters for the SNN solvers of the CMP, Sudoku, and Spin Systems.

**Simulation parameters**				
**CSP**	**Noise populations stimulation (depression)**	**Internal inhibition weights**	**Constraints strength weights**	**External current**
World CMP	10	[−0.08, 0.0]	[−0.08, 0.0]	0.3
Australia CMP	1 (1)	[−1.2, -1.5]	[ 1.2, 1.4]	0.2
Canada CMP	1 (1)	[−1.2, -1.5]	[ 1.2, 1.4]	0.17
Sudoku easy	1 (0)	[−0.08, 0.0]	[−0.08, 0.0]	0.3
Sudoku hard	1 (0)	[−0.08, 0.0]	[−0.08, 0.0]	0.3
AI Escargot	1 (0)	[−0.03, -0.02]	[−0.03, −0.02]	0.3
AF Ring	1 (0)	[−0.2, 0.0]	[−0.2, −0.0]	0.0
Spin 2D lattices	1 (1)	[−0.2, 0.0]	[−0.2, −0.0]	0.0
Spin AF 3D lattice	1 (0)	[−0.2, 0.0]	[−0.2, −0.0]	0.0
Spin FM 3D lattice	1 (0)	[−0.2, 0.0]	[−0.2, −0.0]	0.0

The hard Sudoku puzzle of Figure 2 was previously solved using spiking (Habenschuss et al., 2013) and rate-based (Mostafa et al., 2015b) neural networks with mean solving times of 29 and 153 s, respectively. The solver presented here reduces the mean solving time for this puzzle to 6.36 s implying a considerable improvement in performance for Sudoku neural solvers. The same network parameters were used to solve the three Sudoku puzzles in order to show the relation between the stochastic search and the puzzle difficulty. Clearly, the average time for convergence increases with the difficulty, but more significant is the strong decrease of the success ratio. Thus, to avoid overfitting, a trade-off between exploratory and greedy behavior needs to be found for the problem at hand. The state of the art Sudoku solvers (see for example Norvig, 2009; Dong, 2012) are able to solve puzzles in tens to hundreds of microseconds. Such solvers use backtracking together with deductive methods specific for Sudoku. Consequently, they are not general purpose as the one presented here, it is precisely the specificity what provides their speed-up.

The solution to the map of the territories of Canada, as defined in Figure 1, was presented by D-Wave systems to demonstrate the applicability of their quantum computer. To find the solution they executed a quantum machine instruction which can return 10, 000 samples/s from which ≈25% solved the problem (Headquarters, 2013). This means an effective time to solution of 0.4 ms. The power consumption of the machine is 25 kW and it operates at a temperature of 0.015K. For this same map, our solver uses three SpiNNaker chips each one consuming at most 1 W of power and it finds the solution with a mean time of 0.87 s. Additionally, classical techniques like simulated annealing (Chams et al., 1987), genetic algorithms (Gwee et al., 1993), and tabu search (Dorne and Hao, 1999) as well as the more elaborated state-of-the-art algorithms (Chams et al., 1987; Gwee et al., 1993; Dorne and Hao, 1999; Fotakis et al., 2001; Chiarandini and Stützle, 2002; Galinier and Hertz, 2006; Blöchliger and Zufferey, 2008; Hertz et al., 2008; Ge et al., 2010; Lü and Hao, 2010; Titiloye and Crispin, 2011), solve coloring map problems in time scales ranging from tens of seconds to tens of thousands of seconds and conventionally have a success ratio below 1 for the allocated time. As seen in Figure 4, this is the same order of magnitude for the time that our SNNs needed to solve the coloring map problems of Figure 1.

It is then verified that the solutions found by the SNNs in SpiNNaker are on the order of magnitude of the systems of interest. Our performance is however not competitive with problem-specific solvers which are able to find solutions in a few microseconds. Although such algorithms are extremely fast, they do not perform well if the problem is not solvable by the presumed strategies. If one still desires to find solutions in the order of microseconds, one could resource to accelerated hardware e.g., BrainScales (Schemmel et al., 2010) which runs 10, 000 times faster than real-time (resolution of milliseconds). Unfortunately, these systems are still limited by the number of neurons and synapses they are able to handle. Better performance is also expected from the second generation of SpiNNaker which is currently under development. It is also important to highlight that the NP feature of an algorithm refers to its increasing complexity with the size of the problem, and that the problems presented here correspond to instances of expressly modest sizes. Nevertheless, the number of variables for most problems in robotics and perception have an order of magnitude comparable to that of these CSPs.

The main advantage of stochastic search algorithms is that they are general purpose, able to find satisfactory solutions without needing much detail about the specific problem at hand. Moreover, the exploration of solutions to constraint satisfaction situations never seen before is the typical way in which organisms explore the environment and acquire knowledge about it. To build the solvers of the previous section, we have used only the number of variables, domain size, and constraints list, nevertheless the network showed good performance. Thus if a system of SNNs is able to collect this kind of information from its environment, it will easily take beneficial decisions.

Future work involves the extension of the framework to solve optimization problems where the constraints are defined by inequalities (e.g., to solve the traveling salesman problem or to find the minimum energy configuration of a spin glass), or other more general non-linear constraints. The main concern with such class of problems is that the network is not able to recognize the best option among all the configurations that satisfy the constraints. This is a typical disadvantage of stochastic search algorithms. Thus, the network may visit the optimal solution but will not stay in it. To achieve convergence more complex techniques or even non-stochastic strategies could be needed. The techniques from nonlinear programming could guide the improvement of SNN solvers in decision making under more complex constraints.

In summary, we have presented a neuromorphic implementation of SNNs stimulated with Poisson spike sources which solve CSPs. The network dynamics implements a stochastic search over the problem's space of states which, with an adequate choice of parameters, is able to converge to a stable configuration (or set of configurations) that satisfy all the constraints. A satisfactory performance was found and further research is needed for CSPs defined by more complex constraints. Furthermore, we presented a software framework to explore new strategies for stochastic searches with SNNs. The code of the framework and examples presented here is made available at https://github.com/GAFonsecaGuerra/SpiNNakerCSPs.

## Author contributions

GF developed the SpiNNaker SNN-CSP solver, performed and analyzed the simulations and wrote the manuscript. SF provided the initial scripts, supervised the experiments, discussed the results, and reviewed the manuscript.

### Conflict of interest statement

The authors declare that the research was conducted in the absence of any commercial or financial relationships that could be construed as a potential conflict of interest. The reviewer HM and handling Editor declared their shared affiliation.

## Footnotes

^1^A set of yet harder problems form the NP-Hard class of which P, NP, and NP-Complete problems are subsets, though NP-Hard problems are not necessarily NP.

## References

[B1] Aloupis G. Demaine E. D. Guo A. Viglietta G. (2015). Classic nintendo games are (computationally) hard. Theor. Comput. Sci. 586, 135–160. 10.1016/j.tcs.2015.02.037

[B2] Appel K. I. Haken W. (1989). Every Planar Map is Four Colorable, Vol. 98. Providence, RI: American Mathematical Society Providence.

[B3] Barahona F. (1982). On the computational complexity of Ising spin glass models. J. Phys. A Math. Gen. 15, 3241–3253. 10.1088/0305-4470/15/10/028

[B4] Benjamin B. V. Gao P. McQuinn E. Choudhary S. Chandrasekaran A. R. Bussat J. M. (2014). Neurogrid: a mixed-analog-digital multichip system for large-scale neural simulations. Proc. IEEE 102, 699–716. 10.1109/JPROC.2014.2313565

[B5] Blöchliger I. Zufferey N. (2008). A graph coloring heuristic using partial solutions and a reactive tabu scheme. Comput. Operat. Res. 35, 960–975. 10.1016/j.cor.2006.05.014

[B6] Chaitin G. J. (1982). Register allocation and spilling via graph coloring. Sigplan Not. 17, 98–101.

[B7] Chams M. Hertz A. De Werra D. (1987). Some experiments with simulated annealing for coloring graphs. Eur. J. Oper. Res. 32, 260–266.

[B8] Chiarandini M. Stützle T. (2002). An application of iterated local search to graph coloring problem, in Proceedings of the Computational Symposium on Graph Coloring and its Generalizations (Ithaca, NY), 112–125.

[B9] Churchland P. S. (2008). The impact of neuroscience on philosophy. Neuron 60, 409–411. 10.1016/j.neuron.2008.10.023 18995813

[B10] Clarke A. M. Friedrich J. Tartaglia E. M. Marchesotti S. Senn W. Herzog M. H. (2015). Human and machine learning in non-markovian decision making. PLoS ONE 10:e0123105. 10.1371/journal.pone.0123105 25898139 PMC4405578

[B11] Cobham A. (1965). The intrinsic computational difficulty of functions, in Logic, Methodology and Philosophy of Science, Proceedings of the 1964 International Congress, Studies in Logic and the Foundations of Mathematics, ed Yehoshua B.-H. (Jerusalem: North-Holland Publishing Company), 24–30.

[B12] Colbourn C. J. (1984). The complexity of completing partial latin squares. Discrete Appl. Math. 8, 25–30.

[B13] Cook S. A. (1971). The complexity of theorem-proving procedures, in Proceedings of the Third Annual ACM Symposium on Theory of Computing (Shaker Heights, OH: ACM), 151–158.

[B14] Crair M. C. Bialek W. (1990). Non-boltzmann dynamics in networks of spiking neurons, in Advances in Neural Information Processing Systems (Denver, CO), 109–116.

[B15] Currin A. Korovin K. Ababi M. Roper K. Kell D. B. Day P. J.. (2017). Computing exponentially faster: implementing a non-deterministic universal turing machine using DNA. J. R. Soc. Interface 14:20160990. 10.1098/rsif.2016.0990 28250099 PMC5378132

[B16] Dailey D. P. (1980). Uniqueness of colorability and colorability of planar 4-regular graphs are NP-complete. Discrete Math. 30, 289–293.

[B17] Dong Z. Y. (2012). Zsolver. Available online at: http://forum.enjoysudoku.com/software/ZSolver1.0.zip

[B18] Dorne R. Hao J.-K. (1999). Tabu search for graph coloring, T-colorings and set T-colorings, in Meta-Heuristics, eds Voß S. Martello S. Osman I. H. Roucairol C. (New York, NY: Springer), 77–92.

[B19] Edwards S. F. Anderson P. W. (1975). Theory of spin glasses. J. Phys. F Met. Phys. 5:965.

[B20] Ercsey-Ravasz M. Toroczkai Z. (2012). The chaos within sudoku. Sci. Rep. 2:725. 10.1038/srep00725 23061008 PMC3468838

[B21] Felgenhauer B. Jarvis F. (2005). Enumerating Possible Sudoku Grids. Available online at: http://www.afjarvis.staff.shef.ac.uk/sudoku/sudoku.pdf

[B22] Fortnow L. (2009). The status of the p versus np problem. Commun. ACM 52, 78–86. 10.1145/1562164.1562186

[B23] Fotakis D. Likothanassis S. Stefanakos S. (2001). An evolutionary annealing approach to graph coloring, in Applications of Evolutionary Computing (Como), 120–129.

[B24] Furber S. (2012). To build a brain. IEEE Spectr. 49, 44–49. 10.1109/MSPEC.2012.6247562

[B25] Furber S. (2016). Large-scale neuromorphic computing systems. J. Neural Eng. 13:051001. 10.1088/1741-2560/13/5/051001 27529195

[B26] Furber S. B. (2016). Brain-inspired computing. IET Comput. Digit. Tech. 10, 299–305. 10.1049/iet-cdt.2015.0171

[B27] Furber S. B. Galluppi F. Temple S. Plana L. A. (2014). The SpiNNaker project. Proc. IEEE 102, 652–665. 10.1109/JPROC.2014.2304638

[B28] Furber S. B. Lester D. R. Plana L. A. Garside J. D. Painkras E. Temple S. (2013). Overview of the SpiNNaker system architecture. IEEE Trans. Comput. 62, 2454–2467. 10.1109/TC.2012.142

[B29] Galinier P. Hertz A. (2006). A survey of local search methods for graph coloring. Comput. Oper. Res. 33, 2547–2562. 10.1016/j.cor.2005.07.028

[B30] Gary M. R. Johnson D. S. (1979). Computers and Intractability: A Guide to the Theory of NP-Completeness. New York, NY: W. H. Freeman & Co.

[B31] Ge F. Wei Z. Tian Y. Huang Z. (2010). Chaotic ant swarm for graph coloring, in IEEE International Conference on Intelligent Computing and Intelligent Systems (ICIS), 2010, Vol. 1 (Xiamen), 512–516.

[B32] Goodman D. Khan B. Khan S. Luján M. Watson I. (2013). Software transactional memories for scala. J. Parallel Distrib. Comput. 73, 150–163. 10.1016/j.jpdc.2012.09.015

[B33] Grymel M. Furber S. B. (2011). A novel programmable parallel CRC circuit. IEEE Trans. Very Large Scale Integr. Syst. 19, 1898–1902. 10.1109/TVLSI.2010.2058872

[B34] Gwee B.-H. Lim M.-H. Ho J.-S. (1993). Solving four-colouring map problem using genetic algorithm, in First New Zealand International Two-Stream Conference on Artificial Neural Networks and Expert Systems, 1993 (Dunedin), 332–333.

[B35] Habenschuss S. Jonke Z. Maass W. (2013). Stochastic computations in cortical microcircuit models. PLoS Comput. Biol. 9:e1003311. 10.1371/journal.pcbi.1003311 24244126 PMC3828141

[B36] Headquarters C. (2013). Programming with D-Wave: Map Coloring Problem. Palo Alto, CA: D-Wave Systems, Inc.

[B37] Hertz A. Plumettaz M. Zufferey N. (2008). Variable space search for graph coloring. Discrete Appl. Math. 156, 2551–2560. 10.1016/j.dam.2008.03.022

[B38] Hinton G. E. Sejnowski T. J. (1983). Optimal perceptual inference, in Proceedings of the IEEE conference on Computer Vision and Pattern Recognition (New York, NY), 448–453.

[B39] Hopcroft J. E. Motwani R. Ullman J. D. (2006). Introduction to Automata Theory, Languages, and Computation, 3rd Edn. Harlow: Addison-Wesley.

[B40] Hopfield J. J. (1982). Neural networks and physical systems with emergent collective computational abilities. Proc. Natl. Acad. Sci. U.S.A. 79, 2554–2558. 6953413 10.1073/pnas.79.8.2554 PMC346238

[B41] Hopfield J. J. Tank D. W. (1985). “Neural” computation of decisions in optimization problems. Biol. Cybern. 52, 141–152. 4027280 10.1007/BF00339943

[B42] Jonke Z. Habenschuss S. Maass W. (2016). Solving constraint satisfaction problems with networks of spiking neurons. Front. Neurosci. 10:118. 10.3389/fnins.2016.00118 27065785 PMC4811945

[B43] Karp R. M. (1972). Reducibility among combinatorial problems, in Complexity of Computer Computations, ed Miller R. E. (New York, NY: Springer; Plenum Press), 85–103.

[B44] Lü Z. Hao J.-K. (2010). A memetic algorithm for graph coloring. Eur. J. Oper. Res. 203, 241–250. 10.1016/j.ejor.2009.07.016

[B45] Maass W. (1995). On the computational power of noisy spiking neurons, in Proceedings of the 8th International Conference on Neural Information Processing Systems (Denver, CO; Cambridge, MA: MIT Press), 211–217.

[B46] Maass W. (1996). Lower bounds for the computational power of networks of spiking neurons. Neural Comput. 8, 1–40.

[B47] Maass W. (1997). Networks of spiking neurons: the third generation of neural network models. Neural Netw. 10, 1659–1671.

[B48] Malaka R. Buck S. (2000). Solving nonlinear optimization problems using networks of spiking neurons, in Proceedings of the IEEE-INNS-ENNS International Joint Conference on Neural Networks. IJCNN 2000. Neural Computing: New Challenges and Perspectives for the New Millennium, Vol. 6 (Como), 486–491.

[B49] Maruoka A. (2011). Concise Guide to Computation Theory. London: Springer Science & Business Media.

[B50] Mead C. (1990). Neuromorphic electronic systems. Proc. IEEE 78, 1629–1636.

[B51] Merolla P. A. Arthur J. V. Alvarez-Icaza R. Cassidy A. S. Sawada J. Akopyan F.. (2014). A million spiking-neuron integrated circuit with a scalable communication network and interface. Science 345, 668–673. 10.1126/science.1254642 25104385

[B52] Mostafa H. Müller L. K. Indiveri G. (2013). Recurrent networks of coupled winner-take-all oscillators for solving constraint satisfaction problems, in Advances in Neural Information Processing Systems (Lake Tahoe, NV), 719–727.

[B53] Mostafa H. Müller L. K. Indiveri G. (2015a). An event-based architecture for solving constraint satisfaction problems. Nat. Commun. 6:8941. 10.1038/ncomms9941 26642827 PMC4686837

[B54] Mostafa H. Müller L. K. Indiveri G. (2015b). Rhythmic inhibition allows neural networks to search for maximally consistent states. Neural Comput. 27, 2510–2547. 10.1162/NECO_a_00785 26496042

[B55] Norvig P. (2009). Solving every sudoku puzzle. Available online at: http://norvig.com/sudoku.html

[B56] Painkras E. Plana L. A. Garside J. Temple S. Galluppi F. Patterson C. (2013). SpiNNaker: a 1-w 18-core system-on-chip for massively-parallel neural network simulation. IEEE J. Solid-State Circ. 48, 1943–1953. 10.1109/JSSC.2013.2259038

[B57] Russell S. Norvig P. (2009). Artificial Intelligence: A Modern Approach, 3rd Edn. Upper Saddle River, NJ: Pearson.

[B58] Schemmel J. Briiderle D. Griibl A. Hock M. Meier K. Millner S. (2010). A wafer-scale neuromorphic hardware system for large-scale neural modeling, in Proceedings of 2010 IEEE International Symposium on Circuits and Systems (Paris), 1947–1950.

[B59] Shannon C. E. (1948). A mathematical theory of communication. Bell Syst. Tech. J. 27, 379–423.

[B60] Stein R. B. (1967). Some models of neuronal variability. Biophys. J. 7, 37–68. 19210981 10.1016/S0006-3495(67)86574-3 PMC1368056

[B61] Titiloye O. Crispin A. (2011). Quantum annealing of the graph coloring problem. Discrete Optim. 8, 376–384. 10.1016/j.disopt.2010.12.001

[B62] Wolfram Research, I (2017). Mathematica, Version 11.1. Champaign, IL.

